# The adjuvant effect of bacterium-like particles depends on the route of administration

**DOI:** 10.3389/fimmu.2023.1082273

**Published:** 2023-01-19

**Authors:** Haruka Sudo, Nagisa Tokunoh, Ayato Tsujii, Sarana Kawashima, Yuta Hayakawa, Hiroki Fukushima, Keita Takahashi, Tetsuo Koshizuka, Naoki Inoue

**Affiliations:** Laboratory of Microbiology and Immunology, Gifu Pharmaceutical University, Gifu, Japan

**Keywords:** bacterium-like particles, oral vaccine, nasal vaccine, adjuvant, *Citrobacter rodentium*, lactic acid bacteria, nasal-associated lymphoid tissues

## Abstract

Direct administration of vaccines to mucosal surfaces, such as *via* oral or nasal vaccination, represents an attractive alternative, or complement, to current parenteral vaccination because it has a potential to induce antigen-specific immunity both at mucosal and systemic tissues. Although bacterium-like particles (BLPs), peptidoglycan structures derived from lactic acid bacteria, have been investigated as a novel adjuvant for oral or nasal vaccines, it remains unclear whether the administration routes differ the adjuvant effect of BLPs. Here, we showed that the adjuvant effect of BLPs from *Lactococcus lactis* NZ9000 is greater with the nasal administration than with the oral administration. We conjugated BLPs with Tir, a virulence factor of *Citrobacter rodentium*, as a model adjuvant-antigen complex, and found that nasal, but not oral, immunization of mice with BLP-Tir induced robust antigen-specific IgA responses at the respiratory and intestinal mucosa, IgG2b-skewed systemic responses, and Th17 cellular responses. As one of the underlying mechanisms, we demonstrated that the nasal administration has a greater delivery efficiency (~1,000-fold) of the BLPs-conjugated antigens to mucosal-associated lymphoid tissues than the oral administration. Furthermore, the nasal, but not oral, administration of BLP-Tir elicited robust innate immune responses that were characterized by the expression of various pro-inflammatory cytokines and chemokines in the mucosal-associated lymphoid tissues. Considering these findings together, we anticipate that BLPs can be an attractive novel adjuvant for nasal vaccines targeting not only respiratory but also gastrointestinal infectious diseases.

## Introduction

Mucosal tissues are the main portal *via* which many pathogens invade the host body. Therefore, mucosal immunity is the pivotal first line of defense against invading pathogens. Parenteral vaccination induces systemic immune responses and protects the host from pathogens and toxins. However, as parenteral vaccination induces only limited mucosal immunity, it is not effective in preventing pathogen infection of mucosal tissues, resulting in the primary growth of such pathogens and their spread to susceptible others. In contrast, direct administration of vaccines to mucosal surfaces, such as *via* oral or nasal vaccination, has the potential to induce both mucosal and systemic immune responses (1). Thus, mucosal vaccination represents an attractive alternative, or complement, to parenteral vaccination.

Most licensed mucosal vaccines are live-attenuated vaccines (2), which possess several potential risks, such as uncontrolled replication in immunocompromised hosts, vaccine-associated secondary infection, and development of virulent vaccine-derived strains in the vaccinee (3). Subunit vaccines using recombinant antigens represent a highly safe vaccine strategy, but the addition of an adjuvant is required to induce sufficient immunity (4). However, a safe and potent adjuvant capable of inducing mucosal immunity has not yet been put into practical use.

Bacterium-like particles (BLPs) consist of a peptidoglycan layer containing no proteins or nucleic acids that is prepared by boiling lactic acid bacteria in an acidic solution (5). BLPs activate the innate immune system mainly *via* Toll-like receptor (TLR) 2 and have been studied as a novel adjuvant for vaccines against various infectious diseases (6–9). To date, most studies on BLP-based vaccine systems have been conducted by intranasal administration. Although a few studies have investigated the adjuvant effect of orally administrated BLPs (10–12), it is unclear whether the adjuvant activity of BLPs differs between nasal and oral administration.

Here, we investigated the effect of administration route on the adjuvant activities of BLPs. To this end, immune responses were evaluated in mice immunized with a model antigen: here, we used Tir (translocated-intimin receptor), an effector protein involved in the intestinal epithelial cell adhesion of the mouse colitogenic pathogen *Citrobacter rodentium* (*C. rodentium*). We found that BLP conjugated with Tir showed potent adjuvant activity only when it was administered *via* the nasal route. Furthermore, we scrutinized the administration route-dependent differences in distribution of BLPs in lymphoid tissues and BLP-induced gene expression.

## Materials and methods

### Preparation of BLPs

*Lactococcus lactis* (*L. lactis*) MG1363-derived NZ9000 (Mobitec) was cultured overnight in GM17 medium (M17 containing 5% D-glucose). After centrifugation of the culture, the pellet was washed with phosphate-buffered saline (PBS). The pellet was suspended in 1 M H_2_SO_4_ (pH 1.0) and heated in a water bath at 100°C for 30 min. The heated suspension was centrifuged, the supernatant was removed, the pellet was washed for 3 times with PBS, and then resuspended in PBS to prepare BLPs. BLPs were counted using a hemocytometer and stored at -20°C until use.

### Preparation of antigens

The Tir gene fragment (encoding amino acids 257 to 409 of full-length Tir) and the NanoLuc gene fragment was amplified by PCR using *C. rodentium* ATCC51459 genome DNA and pNL1.1 (Promega) as a template, respectively. The fragments were then cloned into the downstream of the GST gene of the pGEX vector to construct the Tir or NanoLuc expression plasmid (pGEX-Tir and pGEX-NanoLuc). The LysM motif of the AcmA gene of *L. lactis* NZ9000 was cloned into pGEX-Tir or pGEX-NanoLuc to construct pGEX-Tir-LysM and pGEX-NanoLuc-LysM expressing the Tir- and NanoLuc-LysM fusion proteins, respectively. pGEX-Tir, -Tir-LysM and -NanoLuc-LysM were transformed into *E. coli* BL21, resulting in BL21/pGEX-Tir, BL21/pGEX-Tir-LysM and BL21/pGEX-NanoLuc-LysM, respectively. BL21/pGEX-Tir, BL21/pGEX-Tir-LysM and BL21/pGEX-NanoLuc-LysM were stimulated with 0.1 mM IPTG to induce the expression of GST-Tir, GST-Tir-LysM and GST-NanoLuc-LysM for 6 h at room temperature, then collected by centrifugation and disrupted with sonication. Debris was removed by centrifugation to obtain soluble fractions containing recombinant GST-Tir, -Tir-LysM and -NanoLuc-LysM. GST-Tir was affinity-purified using Glutathione Sepharose 4B. Purified Tir was dialyzed against PBS and stored at -80°C until use for ELISA and ELISPOT assay.

### Preparation of antigen-bound BLPs

Tir-LysM- and NanoLuc-LysM-bound BLPs (BLP-Tir and BLP-NanoLuc) were prepared by mixing BLPs with the soluble fraction of BL21/pGEX-Tir-LysM or BL21/pGEX-NanoLuc-LysM containing GST-Tir-LysM and -NanoLuc-LysM, respectively, at room temperature for 30 min. The unbound protein was removed by washing 5 times with PBS. The binding of Tir-LysM to the BLPs was confirmed by confocal laser scanning microscopy after immunostaining with anti-Tir serum (13). The amount of Tir-LysM bound to BLPs was quantified by densitometry analysis of CBB-stained gels. The amount of NanoLuc-LysM bound to BLPs was quantified by luciferase assay. BLP-Tir and BLP-NanoLuc were suspended in PBS and stored at -80°C until use.

### Reporter gene assay

TLR2 stimulation was assessed by reporter gene assay using human TLR2-expressing HEK293T cells (kindly provided by Dr. Tsuyoshi Sugiyama). HEK293T cells were transfected with a human TLR2-expressing plasmid, pNF-κB-TA-luc (a NF-κB-luciferase reporter plasmid), and pGL4.73 (constitutive Renila luciferase expression plasmid, internal control) using PEImax transfection reagent (Polysciences). After incubation for 48 h, cells were treated with various concentrations of BLPs or 100 ng/ml of Pam3CSK4 (a synthetic TLR2 ligand) for an additional 24 h. Luciferase activities were measured using the Dual-Glo luciferase assay system (Promega) and GloMax multiplate reader (Promega), according to the manufacturer’s instructions.

### Animal experiments

All animal experiments were approved by the Institutional Animal Care and Use Committee of Gifu Pharmaceutical University and that of Gifu University. Mice were maintained under normal husbandry conditions in the Animal Facilities of Gifu University.

### Immunization

C57BL/6 mice (6-week-old, female, SLC Japan) were light anesthetized by inhalation with isoflurane and intranasally administered with 20 μl/dose of BLP-Tir (containing 2 × 10^9^ BLPs bound with approximately 20 μg of Tir-LysM, 10 μl/each nasal passage). For oral administration, the same amount of BLP-Tir as used for the intranasal administration was suspended in 200 μl/dose of PBS. Administration was performed 3 times at 11- to 12-day intervals for 3 consecutive days (d0-2, d13-15, d27-29). One week after the final administration (d35), blood, feces, BALF (broncho-alveolar lavage fluid), and nasal lavage fluid were collected to measure Tir-specific antibody titers. Sera were prepared from the blood specimens by centrifugation (800 ×*g*, 20 min, 4°C) after clotting. Fecal pellets were homogenized (100 mg/ml) in PBS containing a complete protease inhibitor mixture (Roche Diagnostics) and, after centrifugation (20,000 ×*g*, 10 min, 4°C), the supernatants were collected as BALF. Serum, fecal extracts. The BALF was collected by inserting a cannula into the bronchus, injecting 1 ml of PBS, followed by collection by suction. After centrifugation (800 ×*g*, 20 min, 4°C), the supernatant was collected as BALF. Serum, fecal extract, BALF and nasal lavage fluid specimens were stored at -20°C until use for measurement of antibody titers.

### ELISA

For measurement of the Tir-specific antibody titers by ELISA, 96-well plates (MaxiSorp, Nunc) were coated with 100 ng/well of the purified GST-Tir. Sera, fecal extracts, and BALF diluted with PBST containing 5% skim milk were then added and incubated for 1 h at 37°C. HRP-conjugated polyclonal goat anti-mouse IgG, IgG1, IgG2b, IgG2c, IgG3 and IgA antibodies (all from Southern Biotech) were added and further incubated for 1 h at 37°C. Plates were developed using o-phenylenediamine substrate, the reaction was stopped by adding H_2_SO_4_, and OD_492-650_ values were then measured.

### ELISPOT

Single cell suspensions from the spleen were prepared from immunized mice 30 days after the final immunization by mechanical disruption and red blood cell lysing. Cells were then suspended in CTL-test medium (ImmunoSpot). Plates for IFN-γ/IL-4 double color ELISPOT assay (ImmunoSpot) or for IL-17 ELISPOT assay (ImmunoSpot) were coated overnight at 4°C with the cytokine-specific capture antibodies. Freshly isolated single cell suspensions from the spleen (5 × 10^5^ cells/well) were plated with the purified GST-Tir (30 μg/ml). Following incubation for 24 h in an incubator at 37°C and 5% CO_2_, the spots for IFNγ/IL-4 or IL-17 were developed according to the manufacturer’s instructions. Spot images were captured using a digital microscope (400-CAM057, SANWA-SUPPLY). The number of Tir-specific spot-forming cells (SFC) was counted using Image J (https://imagej.nih.gov/ij/).

### Antigen dynamics after the oral and nasal administration of BLP-antigen complex

Mice were administered with 2 × 10^9^ BLP-NanoLuc orally or intranasally and euthanized 2 or 6 h post-administration. The NALT (nasal-associated lymphoid tissue), PPs (Peyer's patches), mesenteric lymph nodes (mLNs), and the spleen were excised, washed in PBS, weighed, and then homogenized in 0.2 ml of PBS. To liberate any NanoLuc sequestered within the biological samples, the homogenates were completely disrupted by sonication. The debris was removed by brief centrifugation (10,000 ×*g*, 1 min, 4°C). The supernatant was mixed with the same volume of Nano-Glo Substrate (Promega), and the luciferase activities were then measured using a GloMax multiplate reader (Promega).

### Quantification of BLP in lymphoid tissues

To label BLPs with FITC, 2.7 × 10^10^ BLPs were mixed with FITC (0.1 mg/ml) in 40 ml of carbonate buffer (0.1 M, pH9.0) overnight at 4°C with rotation. Unbound FITC was removed by centrifugation (10,000 ×*g*, 5 min, 4°C) and the labelled BLPs were washed 3 times with PBS. The FITC-labeled BLPs (BLP-FITC) in tissues were quantified according to the method reported by Eyles et al. (14). Mice were administered with 2 × 10^9^ BLP-FITC orally or nasally and euthanized at 2, 6, or 24 h post-administration. Excised tissues (NALT, PPs, mLNs, and spleen) were washed in PBS, weighed, and homogenized in PBS containing 1% paraformaldehyde (PFA). To homogenize the NALT, PPs, and mLNs, 1 ml of PBS/1% PFA was used, and 10 ml of PBS/1% PFA was used for the spleen. To liberate any BLP-FITC sequestered within the various tissues, the homogenates were further disrupted by sonication. The number of BLPs in disrupted tissue suspensions was enumerated by flow cytometer (FACSverse, BD).

### Cell surface marker analysis of splenocytes

Mice were administered with 2 × 10^9^ BLP-FITC orally or nasally and euthanized at 6 or 24 h post-administration. Single cell suspensions from the spleen were prepared as above. To stain dead cells, cells were treated with APC-Cy7-conjugated FVD780 (eBioscience). The cells were then incubated with TruStain FcX™ (anti-mouse CD16/32) Antibody (Biolegend) to block Fc receptor, followed by incubation with PE-conjugated anti-CD11b (M1/70, Biolegend) and APC-conjugated anti-CD11c (N418, Biolegend). The cells were analyzed by FACSverse (BD). At least 2.5 × 10^5^ live singlet cells were used for the analysis.

### Transcriptome analysis

For transcriptomic analysis, total RNA was extracted from the PPs and NALT of mice at 6 h after the oral or nasal administration with BLP-Tir using RNAiso plus (TAKARA) and further purified using Nucleospin RNA (TAKARA) with DNase I treatment according to the manufacturer’s instructions. RNA library preparation was performed using the NEBNext Ultra RNA Library Prep Kit for Illumina (New England Biolabs) according to the manufacturer’s instructions. After assessing the library quality, sequencing was performed using an Illumina Novaseq 6000 with a read configuration of 150-bp paired-ended reads. A total of at least 20 million reads were generated for each sample. One biological replicate was present for each condition. The DNA Data Bank of Japan accession numbers for the RNA sequencing data reported in this study are DRR403116-DRR403119 (https://www.ddbj.nig.ac.jp/dra/index-e.html). Paired-ended clean reads were aligned to the Mus musculus reference genome (mm10, National Center for Biotechnology Information/University of California, Santa Cruz/Ensembl) using Hisat2 v2.0.5. The featureCounts v1.5.0-p3 program was used to count the read numbers mapped for each gene. Fragments per kilobase per million reads of each gene were calculated based on the length of the gene and read count mapped to the gene. Differential expression analysis between two conditions per group was performed using the DESeq2 R package (1.20.0). Genes with an adjusted P-value <0.05 found by DESeq2 were assigned as differentially expressed. Gene ontology (GO) enrichment analysis of differentially expressed genes (DEGs) was implemented using the clusterProfiler R package. GO terms with a corrected P-value <0.05 were considered significantly enriched by DEGs. mRNA levels of indicated genes were further validated by qRT-PCR using primers listed in **Table 1** (n=3). To analyze the kinetics of gene expression changes, total RNA was extracted from the PPs and NALT before and at 6 h, 1 and 4 days after the 3 consecutive days administration of BLP-Tir and subjected to qRT-PCR (n=3).

**Table 1:** Primer sets used for qRT-PCR.

Gene	Forward primer (5’ to 3’)	Reverse primer (5’ to 3’)
***Ccl20***	ACATACAGACGCCTCTTCC	GTTCACAGCCCTTTTCACC
***Ccr7***	ACAGTCTCTCTAAATGCTCCC	CCCCTACCTTTTTATTCCCATC
***Il-6***	ACAAAGCCAGAGTCCTTCAGAG	TTAGCCACTCCTTCTGTGACTC
***Tnf-a***	AGCCTCTTCTCATTCCTGCTTG	GATGAGAGGGAGGCCATTTG
***Il-17a***	TTTAACTCCCTTGGCGCAAAA	CTTTCCCTCCGCATTGACAC
***Il-17f***	TGCTACTGTTGATGTTGGGAC	AATGCCCTGGTTTTGGTTGAA
***Il-22***	TCAGCTCAGCTCCTGTCACATC	TCCCCAATCGCCTTGATCTC
***Tgf-b***	TTACCTTGGTAACCGGCTGC	CAAGAGCAGTGAGCGCTGAATC
***Saa1***	TGTTCACGAGGCTTTCCAAG	AAAGGCCTCTCTTCCATCAC
***Saa3***	GCAACTACTGGGTTGAGATA	ATTCAGCACATTGGGATG
***Reg3g***	TCAGGTGCAAGGTGAAGTTG	GGCCACTGTTACCACTGCTT
***Gapdh***	GTCGTGGAGTCTACTGGTGTCTTC	GTCATATTTCTCGTGGTTCACACC

### Statistical analysis

Data were analyzed by the One-way ANOVA followed by Dunnett’s or Tukey’s *post hoc* multiple comparison test using GraphPad Prism 8 (GraphPad Software). P values <0.05 were considered significant.

## Results

### Preparation of Tir-bound BLP

We used Tir from *C. rodentium* as a model antigen to compare the adjuvant effect of BLPs between the oral and nasal administration routes. To maximize the effect of BLP, Tir was conjugated with BLPs as a Tir-LysM fusion protein. Any antigen that is fused to the well-known peptidoglycan-binding motif (LysM) to form an antigen-LysM fusion protein can strongly bind to BLPs in a non-covalent manner (5).

**Figure 1:**
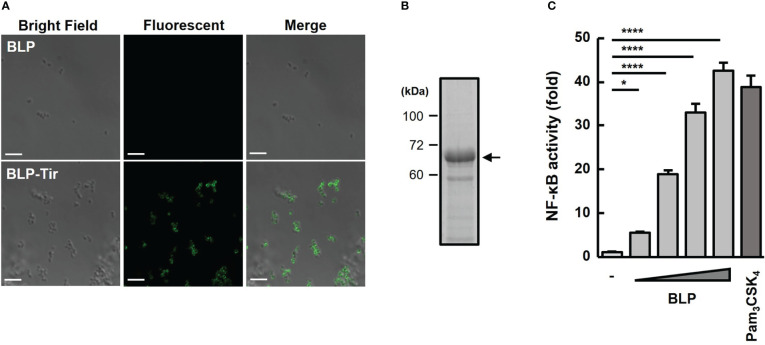
Confirmation of Tir-LysM binding to BLPs. **(A)** Immunofluorescent microscopic images of BLPs alone (upper) and BLP-Tir (Tir-LysM-bound BLP, bottom) labeled with anti-Tir mouse serum and FITC-conjugated anti-mouse IgG. Bars, 5 μm. **(B)** BLP-Tir (2 × 10^9^) was separated by SDS-PAGE, followed by CBB-staining. An arrow indicates 66 kDa BLP-Tir. **(C)** *In vitro* assessment of the TLR2 ligand activity of BLPs. Luciferase expression driven by NF-κB activation in HEK293T-TLR2 reporter cells was measured. Cells were stimulated for 24 h with PBS (-, negative control), BLPs (2, 8, 32 and 128 μg/ml), or Pam_3_CSK_4_ (100 ng/ml, synthetic TLR2 ligand, positive control). Data were expressed as the means ± SD (n = 3). *, p<0.05 and ****, p<0.0001; one-way ANOVA followed by Dunnett’s multiple comparison test.

To examine the binding of BLP and Tir-LysM, BLP alone or BLP-Tir was stained with Tir-specific mouse serum and analyzed by confocal microscopy. Only BLP-Tir was stained with the Tir-specific serum, indicating the binding of BLP and Tir-LysM (**Figure 1A**). To test the binding capacity of BLPs with the Tir-LysM, BLP was incubated with a sufficient amount of Tir-LysM and subjected to SDS-PAGE and CBB staining. Densitometric analysis of the CBB-stained gel showed that the maximum binding capacity of BLPs with Tir-LysM was approximately 20 μg of Tir-LysM per 2 × 10^9^ BLPs (**Figure 1B**). A reporter gene assay using HEK293T-TLR2 reporter cells confirmed that BLPs functioned as a TLR2 ligand (**Figure 1C**).

### The adjuvant effect of BLPs depends on the route of administration

**Figure 2:**
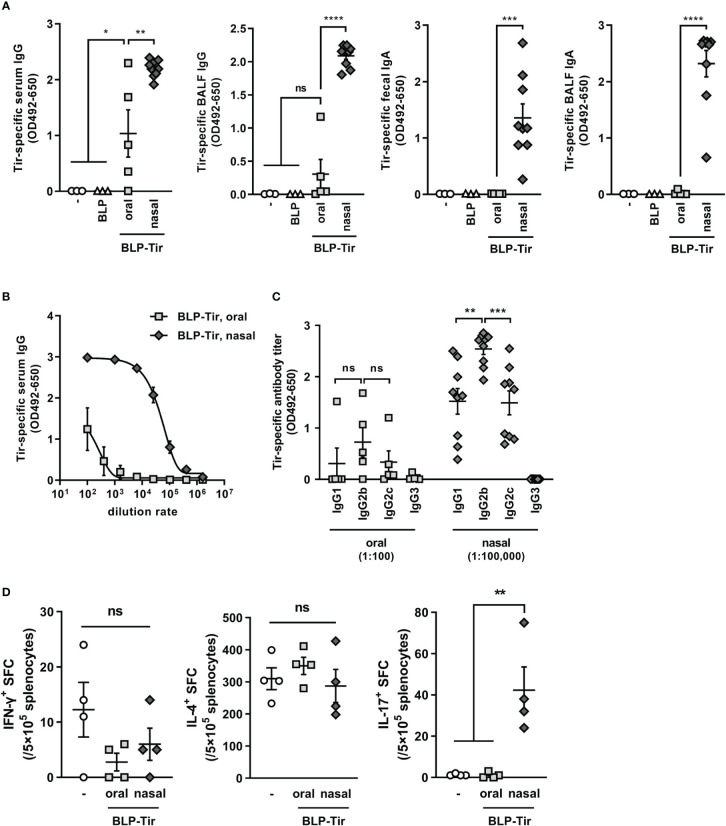
Tir-specific humoral and cellular immune responses induced by oral or nasal administration of BLP-Tir. Mice were administered with BLP-Tir *via* the oral or nasal route for 3 consecutive days for 3 times (d0-2, d13-15, d27-29). **(A)** Tir-specific IgG and IgA in serum, BALF, and fecal extracts collected at d35 were measured. The dilution rate of serum, BALF and fecal extract were 1:100, 1:2, and 1:3, respectively. Data were expressed as the mean ± SEM (n=3-9). Each symbol represents an independent mouse. *, p<0.05; **, p<0.01; ***, p<0.001; and ****, p<0.0001; ns, not significant; one-way ANOVA followed by Tukey’s multiple comparison test. **(B)**, Tir-specific serum IgG antibody titers of the same samples used in **(A)** were measured at various dilution rates. Data were expressed as the mean ± SEM (n=5-9). Some error bars are behind the symbols due to small variations. **(C)**, Tir-specific IgG1, 2b, 2c, and 3 titers. Sera from mice orally administered with BLP-Tir (oral) were diluted 1:100 and those from mice nasally administered with BLP-Tir (nasal) were diluted 1:100,000 (n=5-9). Each symbol represents an independent mouse. **, p<0.01; ***, p<0.001; ns, not significant; one-way ANOVA followed by Tukey’s multiple comparison test. **(D)**, Mice were orally or nasally immunized with BLP-Tir. Splenocytes were isolated 30 days after the last administration. The number of IFN-γ-, IL-4-, and IL-17-spot-forming cells (SFC) calculated from the ELISPOT assay after 30 μg/ml purified Tir stimulation *in vitro*. Data were expressed as the mean ± SEM (n=4). Each symbol represents an independent mouse. **, p<0.01; one-way ANOVA followed by Tukey’s multiple comparison test.

To test the adjuvant effect of BLPs delivered *via* different routes, mice were orally or nasally administered with BLP-Tir, and then Tir-specific humoral and cellular immune responses were assessed. Oral immunization with the BLP-Tir induced a significantly higher level of Tir-specific serum IgG production, but not BALF IgG and fecal or BALF IgA (**Figure 2A**). On the other hand, in mice nasally immunized with BLP-Tir, the Tir-specific mucosal (fecal and BALF) IgA titers as well as serum and BALF IgG titers were increased significantly (**Figure 2A**). The Tir-specific serum IgG titer in nasally immunized mice was 1,000-fold higher than that in orally immunized mice (**Figure 2B**). These results showed that BLPs function effectively as a nasal adjuvant, but their effectiveness as an oral adjuvant is relatively low. Next, we examined the type of antigen-specific immune responses, both in terms of humoral and cellular responses, induced after the oral or nasal administration of BLP-Tir. To examine the type of humoral immune responses, IgG subclasses were analyzed. In mice nasally administered BLP-Tir, IgG2b was increased to the greatest degree, followed by IgG1 and IgG2c, with little increase observed for IgG3 (**Figure 2C**). Although there were no significant differences, the same tendency was observed between the mice administrated nasally and orally (**Figure 2C**). These results indicate that the administration route did not significantly affect the type of immune response induced, at least in terms of the systemic humoral response.

Next, cellular immune responses were assessed by ELISPOT assay using splenocytes. Mice were immunized in the same manner as above, and 30 days after the final administration, isolated splenocytes were stimulated *ex vivo* with recombinant GST-Tir, and the numbers of IFN-γ-, IL-4-, and IL-17-producing cells were measured. The numbers of IFN-γ- and IL-4-producing cells were not increased by either the oral or nasal administration of BLP-Tir in comparison with those for non-immunized mice (**Figure 2D**). However, the number of IL-17-producing cells was significantly increased only in the mice administered BLP-Tir *via* the nasal route (**Figure 2D**). These results indicate that the nasal administration of BLP-Tir mainly induces Th17-type, but not Th1- or Th2-type, cellular immune responses.

### Nasal administration of BLP-antigen complex delivers greater amounts of the antigen to lymphoid tissues than does oral administration

**Figure 3:**
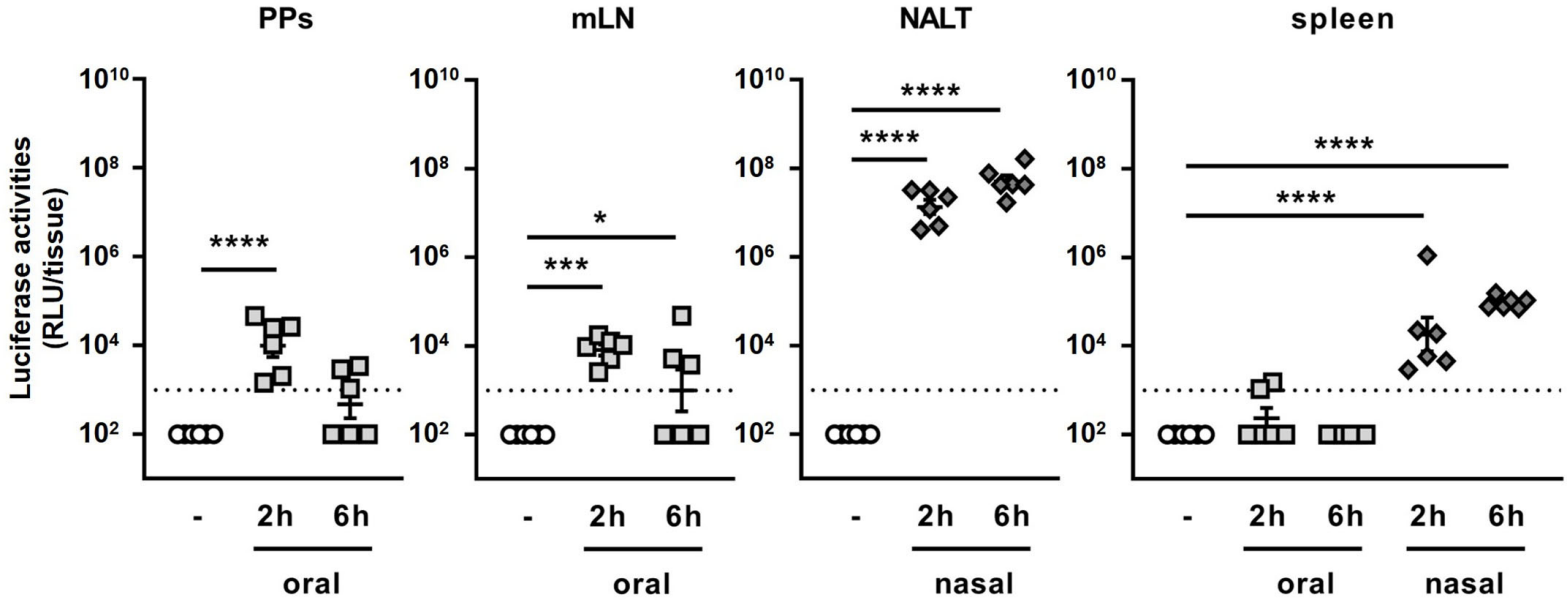
Dynamics of antigen bound to BLPs after oral and nasal administration. The luciferase activities in lymphoid tissues (PPs, mLN, NALT and spleen) at 2 or 6 h after oral or nasal administration of 2 × 10^9^ BLP-NanoLuc. Data were expressed as the mean ± SEM of whole tissue (n=6). Each symbol represents an independent mouse. Dotted line indicates the detection limit. *, p<0.05; ***, p<0.001; and ****, p<0.0001; one-way ANOVA followed by Dunnett’s multiple comparison test.

Next, we attempted to clarify the reason why the adjuvant effect of BLPs greatly differs between the oral and nasal administration routes. One of possible reasons for the weak adjuvant effect of the orally administered BLP-antigen complex is that the BLPs and/or antigen did not reach the gut-associated lymphoid tissue (GALT), the major induction site of antigen-specific immune responses against orally administered antigens (15). To examine whether the antigen reached the mucosal lymphoid tissue after the oral or nasal administration of a BLP-antigen complex, mice were administered BLP-NanoLuc, a luciferase-bound BLP, *via* the oral or nasal route, and the luciferase activities in PPs, mLNs, NALT and the spleen were measured. Significant luciferase activity was detected in the PPs and mLNs, but not in the spleen, at 2 h after the oral administration of BLP-NanoLuc (**Figure 3**). The luciferase activity in the NALT after the nasal administration of BLP-NanoLuc was 1,000 times higher than those observed in the PPs and mLNs after the oral administration of BLP-NanoLuc. Additionally, significant luciferase activity was also detected in the spleen of mice administered BLP-NanoLuc *via* the nasal route (**Figure 3**). In mice orally administered BLP-NanoLuc, decreased in luciferase activities were observed in the PPs and mLNs at 6 h post-administration (**Figure 3**). In contrast, the luciferase activity in the spleen of mice nasally administered BLP-NanoLuc increased over time (**Figure 3**). These results suggest that the oral administration of a BLP-antigen complex delivers only a small amount of antigen to the GALT in an intact form, but the nasal administration of the same BLP-antigen complex results in the delivery of a greater amount of the antigen to mucosal and systemic lymphoid tissues, with the antigen stayed in the tissues for a longer time.

### The number of BLPs reaching the mucosal lymphoid tissue is greater with nasal administration than with oral administration

**Figure 4:**
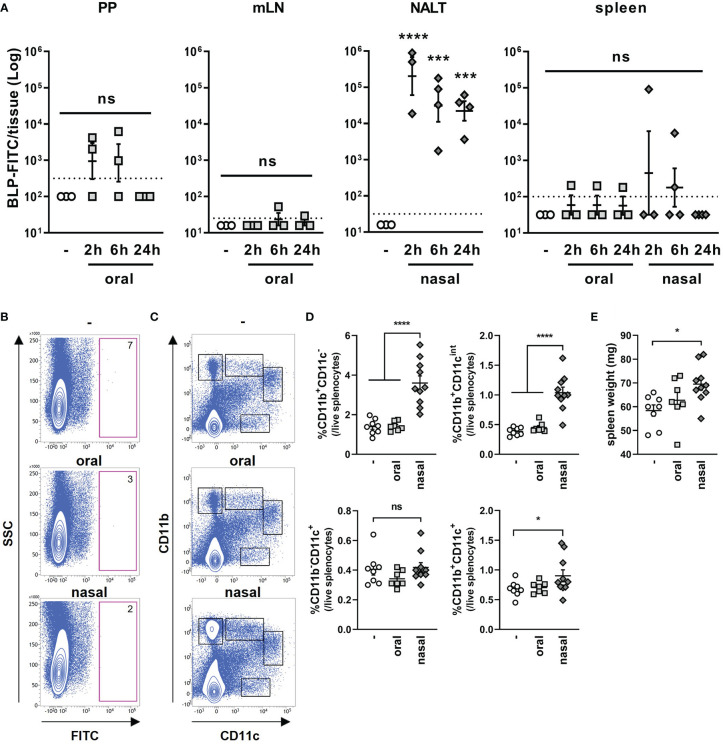
Dynamics of BLPs after oral and nasal administration. **(A)** The number of BLP-FITC conjugates in lymphoid tissues (PPs, mLNs, NALT and spleen) was determined by flow cytometry at 2, 6, or 24 h after oral or nasal administration of 2 × 10^9^ BLP-FITC. Data were expressed as the mean ± SEM (n=3-4). Each symbol represents an independent mouse. Dotted line indicates the detection limit. ***, p<0.001; ****, p<0.0001; one-way ANOVA followed by Tukey’s multiple comparison test. **(B)** Splenocytes were isolated at 6 h after the oral or nasal administration of BLP-FITC. Dead cells were gated out with FVD780 reagent. Representative images of each group are shown (n=3-6). The red frame boxes indicate the number of FITC^+^ cells in approximately 5 × 10^5^ live splenocytes. **(C)** Splenocytes isolated at 24 h after the oral or nasal administration of BLP-FITC were stained with antibodies to CD11b and CD11c. Dead cells were gated out with FVD780 reagent. At least 2.5 × 10^5^ live singlet cells were initially gated. Representative images of each group are shown (n=7-10). **(D)** Percentage of the populations is indicated with boxes in **(C)**. *, p<0.05; ****, p<0.0001; ns, not significant; one-way ANOVA followed by Tukey’s multiple comparison test. **(E)** Spleen weight of mice at 24 h after the oral or nasal administration with BLP-FITC. *, p<0.05; one-way ANOVA followed by Tukey’s multiple comparison test.

Next, we compared the number of BLPs reaching the lymphoid tissue after their oral and nasal administration. To this end, FITC-labeled BLPs (BLP-FITC) were orally or nasally administered to mice, and the number of BLP-FITC conjugates contained in each tissue was examined by flow cytometry. The number of BLP-FITC conjugates detected in the PPs at 2, 6 and 24 h after the oral administration of BLP-FITC varied from below the detection limit to over 10^3^ particles, with no BLP-FITC detectable in the PPs at 24 h post-administration (**Figure 4A**). In the NALT from mice administered nasally, 10^4^-10^6^ BLP-FITC conjugates were detected at 2 h after administration (**Figure 4A**). This represents only 0.1–0.001% of the administered BLP-FITC, but 10–1000 times more than that detected in the PPs after oral administration (**Figure 4A**). Even at 6 or 24 h after the nasal administration, 10^3^-10^5^ BLP-FITC conjugates were still detected in the NALT (**Figure 4A**). However, only small numbers of BLP-FITC were detected in the mLNs and spleen after the oral administration and in the spleen after the nasal administration of BLP-FITC (**Figure 4A**). These results suggest that unlike conjugated antigens, few BLPs themselves migrate to the lymphatics or into systemic circulation regardless of the route of administration.

Previous reports suggest that nano- or micro-particles are taken up by dendritic cells (DCs) and are then translocated to systemic lymphoid tissues (16, 17). To investigate the possibility of BLP translocation *via* DCs to the spleen, BLP-FITC was orally or nasally administered to mice. Splenocytes were then isolated at 6 h post-administration, and the cells containing BLP-FITC was measured. However, no cells were observed to have taken up BLP-FITC in the spleen, suggesting that the BLP-FITC detected in the spleen of some mice may exist in cell-free state (**Figure 4B**). On the other hand, a significant increase in the proportions of CD11b^+^CD11c^-^, CD11b^+^CD11c^int^ and CD11b^+^CD11c^+^ subsets were observed in the spleen of mice at 24 h after the nasal administration of BLP-FITC (**Figures 4C, D**). Furthermore, in the nasally administered mice, an increase in spleen weight was also observed (**Figure 4E**). These results suggested that the small numbers of BLPs translocated to the spleen induce an increase in at least the CD11b^+^ cell subsets in the spleen.

### Nasal, but not oral, administration of BLP-Tir induces robust innate immune responses

**Figure 5:**
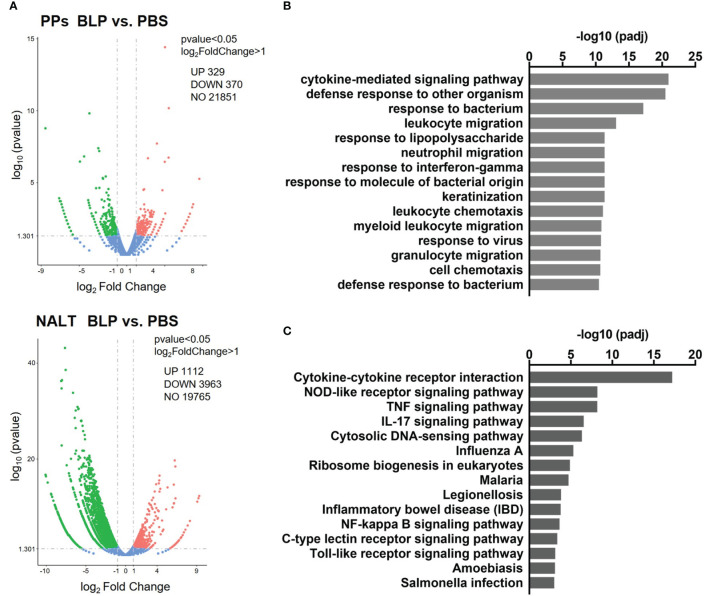
Transcriptome analysis of the mucosal lymphoid tissues. Mice were administered orally or nasally with BLP-Tir or PBS. RNA-seq analysis was performed using total RNA extracted from the PPs of orally administered mice and from the NALT of nasally administered mice at 6 h post-administration (n=1). **(A)** Volcano plots represent DEGs between the PPs (upper) and the NALT (bottom) of PBS and BLP-Tir administered mice. **(B)** Gene ontology (GO) and **(C)**, KEGG pathway analysis of genes upregulated in the NALT of BLP-Tir administered mice compared with those of the PBS administered mice.

To clarify the reaction in the mucosal lymphoid tissue against BLPs, differences in gene expression in the PPs and NALT at 6 h after the oral and nasal administration of BLP-Tir were analyzed by RNA-seq. Oral administration of BLP-Tir induced an increase and decrease in the expression of 329 and 370 genes, respectively, in the PPs (**Figure 5A**; **Supplementary Data 1**). Nasal administration of BLP-Tir induced robust changes in gene expression in the NALT, with the expression of 1,112 genes upregulated and 3,963 genes downregulated (**Figure 5A**; **Supplementary Data 2**). However, no significant GO terms were extracted from the enrichment analysis of the differentially expressed genes (DEGs), with the expression levels of the cytokine gene *Il-22*, acute phase protein gene Saa1, and antimicrobial peptide genes *Defa33*, *Reg3b*, and *Reg3g* all upregulated in the PPs of mice orally administered with BLP-Tir (**Supplementary Data 1**). In contrast, GO enrichment analysis of DEGs in the NALT from nasally administered mice resulted in the extraction of many immune response-related GO terms (**Figure 5B**). KEGG analysis also identified the immune response-related functions of the upregulated genes (**Figure 5C**).

**Figure 6:**
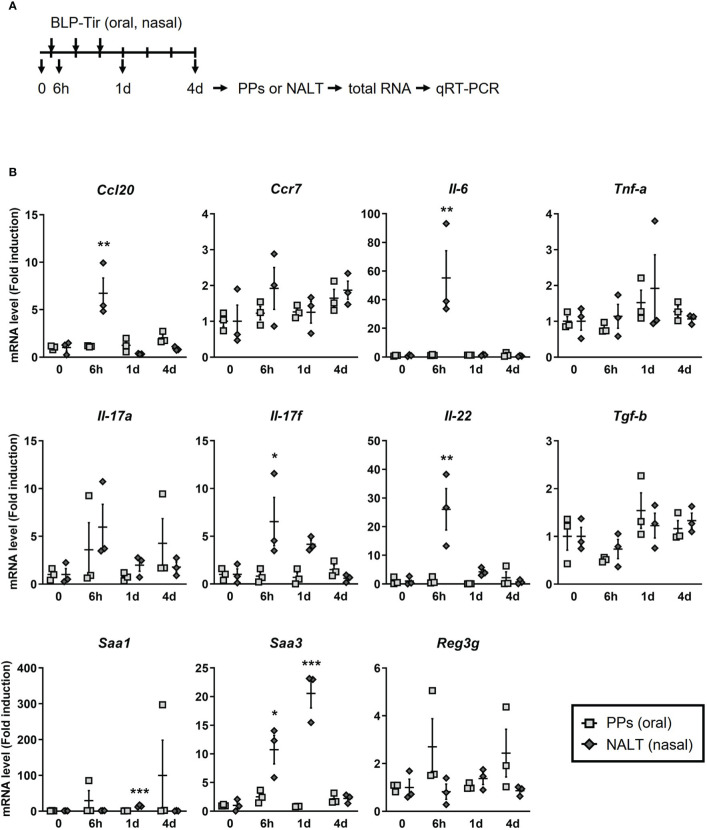
Kinetics of cytokines/chemokines gene expression in mucosal lymphoid tissues. **(A)** Design of the experiment. Mice were administered with BLP-Tir orally or nasally for 3 consecutive days. Total RNA was extracted from the PPs or the NALT from mice administered with BLP-Tir orally or nasally, respectively, before (0) and at 6 h after the 1^st^ administration, and at 1 and 4 days (1d and 4d) after the 3^rd^ administration (n =3/group). **(B)** mRNA levels of the indicated genes were measured by qRT-PCR and expressed as fold induction (means ± SEM, n=3). *Gapdh* was used as an internal control. *, p<0.05; **, p<0.01; ***, p<0.001; one-way ANOVA followed by Dunnett’s multiple comparison test were used to compare means from BLP-Tir administered groups against the control group (0, before administration).

To confirm the RNA-seq data and to examine the kinetics of gene expression, mice were administered BLP-Tir *via* the oral or nasal route for 3 consecutive days, and total RNA was extracted from the PPs or NALT before administration, at 6 h after the first administration, and at 1 and 3 days after the third administration, and then subjected to qRT-PCR (**Figure 6A**). Due to the large variability, no significant increase was observed in the gene expression levels of Saa1 and Reg3g, which were suggested to be upregulated in the PPs after the oral administration of BLP-Tir by RNA-seq analysis (**Figure 6B**). However, the expression levels of these genes clearly increased in some mice, suggesting considerable variability in the PPs among mice in response to orally administered BLP-Tir (**Figure 6B**). These results support the flow cytometric data indicating that a limited/variable number of BLPs reached the PPs after oral administration and suggest that the delivered BLPs elicited weak/transient antimicrobial responses in some mice. Kinetics analysis also showed that gene expression levels of cytokines and chemokines were sharply elevated upon the nasal administration of BLP-Tir, mostly returning to the basal level by 1 day after the 3 consecutive days of administration (**Figure 6B**). *Tnf-a*, *Tgf-b*, and *Reg3g* gene expression levels were not significantly changed in the NALT even after the nasal administration of BLP-Tir (**Figure 6B**). Overall, these results showed that the nasal, but not the oral, administration of BLP-Tir results in the elicitation of strong innate immune responses in the NALT.

## Discussion

In this study, we clearly showed that (1) the adjuvant effect of BLPs is greater with nasal administration than with the oral administration, (2) the nasal administration of BLP-Tir induced antigen-specific antibody responses in the respiratory and intestinal mucosa as well as in systemic tissues, characterized by IgG2b-skewed humoral responses and Th17 cellular responses, (3) the nasal administration of BLP-antigen complexes results in the delivery of much greater numbers of BLPs and larger amounts of antigen to the NALT than those delivered to the GALT after oral administration, (4) the oral administration of BLP-Tir elicits weak antimicrobial responses in some mice while, in contrast, (5) the nasal administration of BLP-Tir elicits robust innate immune responses, including the expression of various pro-inflammatory cytokines and chemokines in the NALT.

Generally, oral vaccination can induce gut-directed mucosal immunity (18). Thus, oral administration is regarded as the preferred administration route for mucosal vaccines targeting gastrointestinal infections. To induce specific immunity, orally administered vaccine needs to overcome numerous hurdles, including pH change, digestive enzymes, thick mucus, peristalsis, limited lymphoid tissue within a large surface area, and tolerance (19–21). Thus, the development of effective adjuvants and novel delivery systems are necessary to overcome those hurdles (2, 18). Our study is in line with adjuvant development using microparticles (22) and demonstrates that there were significant differences in the number of BLPs and amount of antigen reaching the mucosa-associated lymphoid tissues as well as in the degree of activation of the innate immune responses when the same number of BLPs and amount of antigen was administered orally or nasally. As the intranasal administration of BLP-Tir could induce antigen-specific immunity not only to the respiratory mucosa but also to the intestinal mucosa, it is worth further examination of BLP-adjuvanted nasal vaccine systems as an alternative for oral vaccine systems targeting intestinal infections.

To date, most studies on the immune response induced by the nasal administration of BLP-adjuvanted vaccines have focused on the traditional Th1/Th2 balance (10, 12, 23–25) and suggested that BLPs induce Th1-skewed immune responses. Contrary to these previous studies, ELISPOT analysis of splenocytes in this study did not show any increase in IFN-γ- or IL-4- producing cells. This discrepancy may be due to differences in the timing of the analysis after immunization; that is, between 1-2 weeks in the previous studies and 1 month in this study. Our results are in line with studies reporting that mucosal vaccines with synthetic TLR2 ligand adjuvants and respiratory infections with Gram-positive bacteria elicited strong Th17, but not Th1 or Th2, responses (26, 27). IgG subclass analysis showed that BLP-Tir induced IgG2b as the main IgG subclass. Class switching to IgG2b is promoted by TGF-β (28), an essential cytokine for Th17 differentiation (29). Thus, our results suggest that both T and B cells differentiated primarily under the influence of TGF-β in mice nasally administered with BLP-Tir.

We also showed by transcriptome analysis that the expression of various chemokine and cytokine genes increased in the NALT after the intranasal administration of BLP. Chemokines upregulated by the intranasal administration of BLPs included *Cxcl1*, *Cxcl2*, *Cxcl3*, *Cxcl5*, *Cxcl10*, *Ccl2*, *Ccl3*, *Ccl4*, *Ccl7*, and *Ccl20*. In other words, it was shown that the intranasal administration of BLP-Tir facilitated the recruitment of various types of immune cells to the NALT. The enrichment analysis suggested that various signaling pathways, such as NOD-like receptor (NLR)-, TNF-, IL-17-, and cytosolic DNA sensing-signaling, were activated upon the nasal administration of BLP-Tir. Persistent IL-17 production may lead to chronic inflammation (30). However, in phase I clinical trials of BLP-based influenza and respiratory syncytial virus vaccines, no serious adverse effects have been observed (6, 31). These results indicate that the activation of the innate immune responses in the NALT is the main reaction for several hours after the nasal administration of BLPs.

It has been shown in parenteral vaccine systems that particles above 200–500 nm in size cannot enter the lymphatics directly and need to be engulfed by DCs to enter the lymphatics or systemic lymphoid tissues (16, 17). However, BLPs found in the spleen of mice intranasally administered BLP-FITC were not associated with any cells, suggesting that small numbers of BLPs may enter the lymphatics or into circulation in a cell-free form. In addition, the nasal administration of BLPs resulted in increases in CD11b^+^CD11c^-^, CD11b^+^CD11c^int^ and CD11b^+^CD11c^+^ subsets in the spleen. According to studies characterizing spleen cells by CD11b and CD11c expression patterns (32, 33), these cells subsets consist of myeloid cells (including macrophages, neutrophils, and eosinophils), dendritic-like cells, and conventional DCs, respectively. The means by which the increases in these cells contribute to the adjuvant effects of BLPs remains unclear, but our results (*i.e.*, the increase in the CD11b^+^ cell subsets and spleen weight) suggest that nasally administered BLPs activate immune responses not only in the NALT but also in the systemic lymphoid tissues.

Despite a certain amount of antigen as well as BLPs reaching the PPs after oral administration, only limited changes in gene expression occurred. The oral administration of BLP-Tir also resulted in undetectable IgA antibody responses in the intestinal mucosa, supporting the lack of immune responses in the GALT. This hypo-responsiveness in the PPs against orally administered BLP may be due to quantitative and/or functional limitations. In other words, the number of BLPs reaching the PPs was below the threshold sufficient for stimulation of the immune system, and/or the BLPs we used exhibited a poor ability to stimulate the immune system in the PPs. If the quantitative limitation is the main cause of hypo-responsiveness in the PPs, strategies that increase the number of BLPs reaching the PPs, such as M-cell targeting (34), would be useful for enhancing the effect of BLPs as an oral adjuvant. On the other hand, there are some reports suggesting the possibility of a functional limitation of the BLPs used. BLPs prepared from *Lacobacillus rhamnosus* showed a significant adjuvant effect when administered orally with live rotavirus vaccine or hepatitis E antigen (11). The activity to stimulate the immune system depends on the strain used for BLP preparation (12, 35). Thus, screening of strains and elucidation of the mechanisms underlying the differences in stimulatory activities among strains are required for the development of highly effective BLP-based oral vaccines. Of course, immunogenicity is also affected by the properties of the antigen itself (10), it is also necessary to verify the adjuvant effect of BLPs across a wider range of antigens.

In conclusion, the adjuvant effects of BLPs greatly depend on the administration route. Intranasal, but not oral, administration of BLPs was able to induce robust immunity not only in the respiratory mucosa but also in the intestinal mucosa.

## Data availability statement

The datasets presented in this study can be found in online repositories. The names of the repository/repositories and accession number(s) can be found in the article/**Supplementary Material**.

## Ethics statement

The animal study was reviewed and approved by Animal Care and Use Committees of Gifu Pharmaceutical University and Gifu University.

## Author contributions

KT, NI, and TK contributed to conception and design of the study. KT and SK contributed to methodology. HS, NT, YH, HF, KT performed data curation. AT and SK contributed to data validation. KT wrote the first draft of the manuscript. All authors contributed to the article and approved the submitted version.
